# A descriptive assessment of the National Institute of Public Health’s contribution to the COVID-19 response in Cambodia,  2020–2021

**DOI:** 10.5365/wpsar.2023.14.1.974

**Published:** 2023-03-22

**Authors:** Srean Chhim, Wuddhika In Vong, Kimsorn Pa, Chanboroth Chhorn, Tambri Housen, Amy Elizabeth Parry, Wim Van Damme, Por Ir, Chhorvann Chhea

**Affiliations:** a National Institute of Public Health, Phnom Penh, Cambodia.; b University of Newcastle, Newcastle, New South Wales, Australia.; c Australian National University, Canberra, Australian Capital Territory, Australia.; d Institute of Tropical Medicine, Antwerp, Belgium.

## Abstract

**Objective:**

This paper examines the contributions made by the National Institute of Public Health to Cambodia’s response to the coronavirus disease (COVID-19) pandemic during 2020–2021.

**Methods:**

The activities conducted by the Institute were compared with adaptations of the nine pillars of the World Health Organization’s 2020 COVID-19 strategic preparedness and response plan. To gather relevant evidence, we reviewed national COVID-19 testing data, information about COVID-19-related events documented by Institute staff, and financial and technical reports of the Institute’s activities.

**Results:**

The main contributions the Institute made were to the laboratory pillar and the incident management and planning pillar. The Institute tested more than 50% of the 2 575 391 samples for severe acute respiratory syndrome coronavirus 2 (SARS-CoV-2) testing and provided technical advice about establishing 18 new laboratories for SARS-CoV-2 testing in the capital city of Phnom Penh and 11 provinces. The Institute had representatives on many national committees and coauthored national guidelines for implementing rapid COVID-19 testing, preventing transmission in health-care facilities and providing treatment. The Institute contributed to six other pillars, but had no active role in risk communication and community engagement.

**Discussion:**

The Institute’s support was essential to the COVID-19 response in Cambodia, especially for laboratory services and incident management and planning. Based on the contributions made by the Institute during the COVID-19 pandemic, continued investment in it will be critical to allow it to support responses to future health emergencies in Cambodia.

The coronavirus disease (COVID-19) pandemic has impacted people’s well-being globally; Cambodia was no exception. (*1*) The first case of COVID-19 in Cambodia was detected on 27 January 2020. By 31 December 2021, the country had recorded  120 487 cases and 3012 deaths, with a case–fatality rate of 2.5%. (*2*) During the 305 days after the first case of infection with severe acute respiratory syndrome coronavirus 2 (SARS-CoV-2) was detected, there were only 308 cases, the majority of which were imported, and there were no deaths. (*2*) This suggests that  SARS-CoV-2 was contained early on during the pandemic in Cambodia. Experts believed that this low number of cases was due to the early implementation of stringent public health and social measures, such as border and school closures, the cancellation of public events and gatherings, extensive mass testing and intensive contact tracing. (*3*) With the arrival of the SARS-CoV-2 vaccine in February 2021, Cambodia was among the few countries to achieve more than 90% vaccination coverage (i.e. two doses or a complete series of doses) of its population aged 12 years and older by September 2021. (*2*)

The mission of Cambodia’s National Institute of Public Health (NIPH) is to be the leading public health institute in the country. During the past 10 years, before the COVID-19 pandemic, NIPH committed to building its workforce to fulfil this mission by providing opportunities for staff to lead research projects and facilitating staff attendance at short- and long-term capacity-building training activities abroad. More recently, NIPH provided opportunities for staff to be directly involved in responding to the COVID-19 pandemic. NIPH has three specialized technical units: the School of Public Health, the Health Systems Research and Policy Support Center and the National Reference Laboratory. The School of Public Health was founded in 2007 and offers master’s degrees in public health, epidemiology, human nutrition, hospital administration, and health and community development. The Health Systems Research and Policy Support Center promotes evidence-based health systems policies and governance by conducting research and translating findings into policies or actions. The National Reference Laboratory promotes and strengthens the quality of laboratory services for public health, as well as housing a biosafety level 2-plus facility that is capable of conducting molecular surveillance, using methods such as polymerase chain reaction (PCR)-based amplification and next-generation sequencing, to detect pathogenic or infectious organisms posing moderate health hazards.

NIPH was involved in many aspects of the COVID-19 response in Cambodia, and this descriptive assessment documents its contributions.

## Methods

This descriptive assessment examines the contributions made by NIPH to the COVID-19 response in Cambodia between 1 January 2020 and 31 December 2021 by comparing it with the country’s adaptations of the nine pillars of the World Health Organization’s (WHO’s) 2020 COVID-19 strategic preparedness and response plan. (*4*) The nine pillars were adapted and used in Cambodia’s master plan for its COVID-19 response as (i) incident management and planning, (ii) laboratory services, (iii) surveillance, (iv) points of entry, (v) rapid response teams, (vi) infection prevention and control, (vii) case management and continuity of essential services, (viii) logistics, procurement and supply management and (ix) risk communication and community engagement  (**Table 1**). (*4*)

**Table 1:** The nine pillars of the World Health Organization’s 2020 COVID-19 strategic preparedness and response plan (*1*) as adapted by the Cambodia National Institute of Public Health and how the Institute supported each pillar

Pilar	Description	Roles of NIPH
Incident management and planning	Ability of the government to provide guidance and planning assumptions, and make appropriate modifications to laws or regulations at all levels and sectors to enable an effective response	NIPH had representation on six national subcommittees, as well as on Ministry of Health committees; provided technical advice and coauthored four national guidelines and policies addressing rapid testing for COVID-19, conducting vaccination campaigns, preventing transmission in health-care facilities and providing treatment.
Laboratory services	Ability to perform SARS-CoV-2 testing rapidly without the need to ship specimens overseas	NIPH’s laboratory tested 50% of all samples for SARS-CoV-2 and provided technical advice to help establish 18 new laboratories for this testing.
Surveillance	Ability to detect cases and report to global surveillance databases	NIPH tested 4 636 samples for the national severe acute respiratory infection and influenza-like illness surveillance systems, collaborated to establish COVID-19 testing sites throughout Phnom Penh and produced 31 reports that used epidemiological and laboratory data and that were distributed to stakeholders.
Points of entry	Ability to detect cases, isolate them and quarantine contacts properly at the points of entry (international borders)	NIPH collected specimens at the Phnom Penh International Airport and trained 238 trainers in specimen collection, COVID-19 case management and the fundamentals of isolation and quarantine.
Rapid response teams	Teams were created to investigate suspected COVID-19 cases and initiate treatment, if appropriate	NIPH trained 504 rapid response team trainers in COVID-19 responses and contact tracing.
Infection prevention and control	Ensure health-care workers are protected from infection with SARS-CoV-2 during amplification events in health-care facilities, such as while providing testing and care	NIPH coauthored the standard operating procedures for COVID-19 vaccination and also information about prevention and control of COVID-19 transmission at health facilities and clinics in Cambodia.
Case management and continuity of essential services	Designate referral facilities to care for patients with SARS-CoV-2 and ensure that essential services for other patients are continued	NIPH in collaboration with its partners, trained 1 497 trainers, including health-care staff and volunteers, to build capacity to care for COVID-19 patients.
Logistics, procurement and supply management	Ability to communicate rapidly, regularly and transparently with the population	In 2021, NIPH prepared testing packages for mobile testing teams that were used to collect 735 396 specimens in Phnom Penh.
Risk communication and community engagement	NIPH did not play a role in supporting activities associated with this pillar.	NIPH did not play a role in supporting activities associated with this pillar.

COVID-19: coronavirus disease; NIPH: National Institute of Public Health; SARS-CoV-2: severe acute respiratory syndrome coronavirus 2.

We conducted secondary analyses of national COVID-19 testing data owned by the Inter-ministerial Committee to Combat COVID-19 that was managed by the Communicable Disease Control Department (CDCD). We conducted a desk review of COVID-19 policies and guidelines developed during the pandemic to identify those authored by NIPH and reviewed training reports related to COVID-19 that were obtained from NIPH’s accounting team to assess the number of people trained and the topics of the trainings. Therefore, the findings reflect an internal audit of the roles of and activities conducted by NIPH staff and should be interpreted with this in mind.

## Results

This descriptive assessment of the roles of and activities conducted by NIPH staff as part of the COVID-19 response showed that NIPH was involved in actions that supported all but one of the pillars – that is, NIPH was not involved in risk communication and community engagement (**Table 1**).

### Pillar i: incident management and planning

During Cambodia’s COVID-19 response, the Prime Minister was the lead incident manager, with high-level committees providing advice. The high-level committees included the Provincial COVID-19 Committee, chaired by the Provincial Governors, which monitored responses in each province; the High-level Ministry of Health Task Force, which mobilized resources; and the Technical Working Group, led by Cambodia’s CDCD with technical support from governmental and nongovernmental stakeholders, which provided technical advice about all aspects of the COVID-19 emergency response.

The COVID-19 response in Cambodia was flexible and continually evolving. To manage the increase in COVID-19 cases in 2021, the Prime Minister issued six strategies:

Strategy 1 – prevent imported cases of Delta variant SARS-CoV-2;
Strategy 2 – decrease the number of areas considered to be at high risk (as defined by the Cambodian government) and prevent new infections;
Strategy 3 – improve recovery rates and reduce the case–fatality rate;
Strategy 4 – strengthen the identification of cases and contacts;
Strategy 5 – improve the handling of the bodies of those who died from COVID-19 while transferring them for cremation; and
Strategy 6 – boost the vaccination rate.

The government created 10 subcommittees to implement these strategies, which were chaired by the Inter-ministerial Committee.

To aid in incident management and planning, NIPH provided technical advice and developed guidelines and policies. NIPH experts took part in several subcommittees, including the Subcommittees for:

Evaluation, Planning and Strategy;
Management at Points of Entry and Quarantine;
Rapid Response and Investigation;
Technical Advice and Treatment;
Laboratory Services; and
Education, Training and Public Affairs.

NIPH was also an active member of the High-level Ministry of Health Task Force, the Technical Working Group and the Committee for Vaccination. As a member of these committees, NIPH contributed to shaping the national COVID-19 response. NIPH experts coauthored four national guidelines. The policies and guidelines coauthored by NIPH included the:

guideline for the prevention and control of COVID-19 transmission in health facilities and clinics in Cambodia (July 2020);
guidelines for the use of rapid tests for COVID-19 in private health services (May 2021);
standard operating procedures for COVID-19 vaccination (June 2021); and
fourth version of the clinical treatment protocol for COVID-19 (Jan 2022).

### Pillar ii: laboratory services

**Fig. 1:**
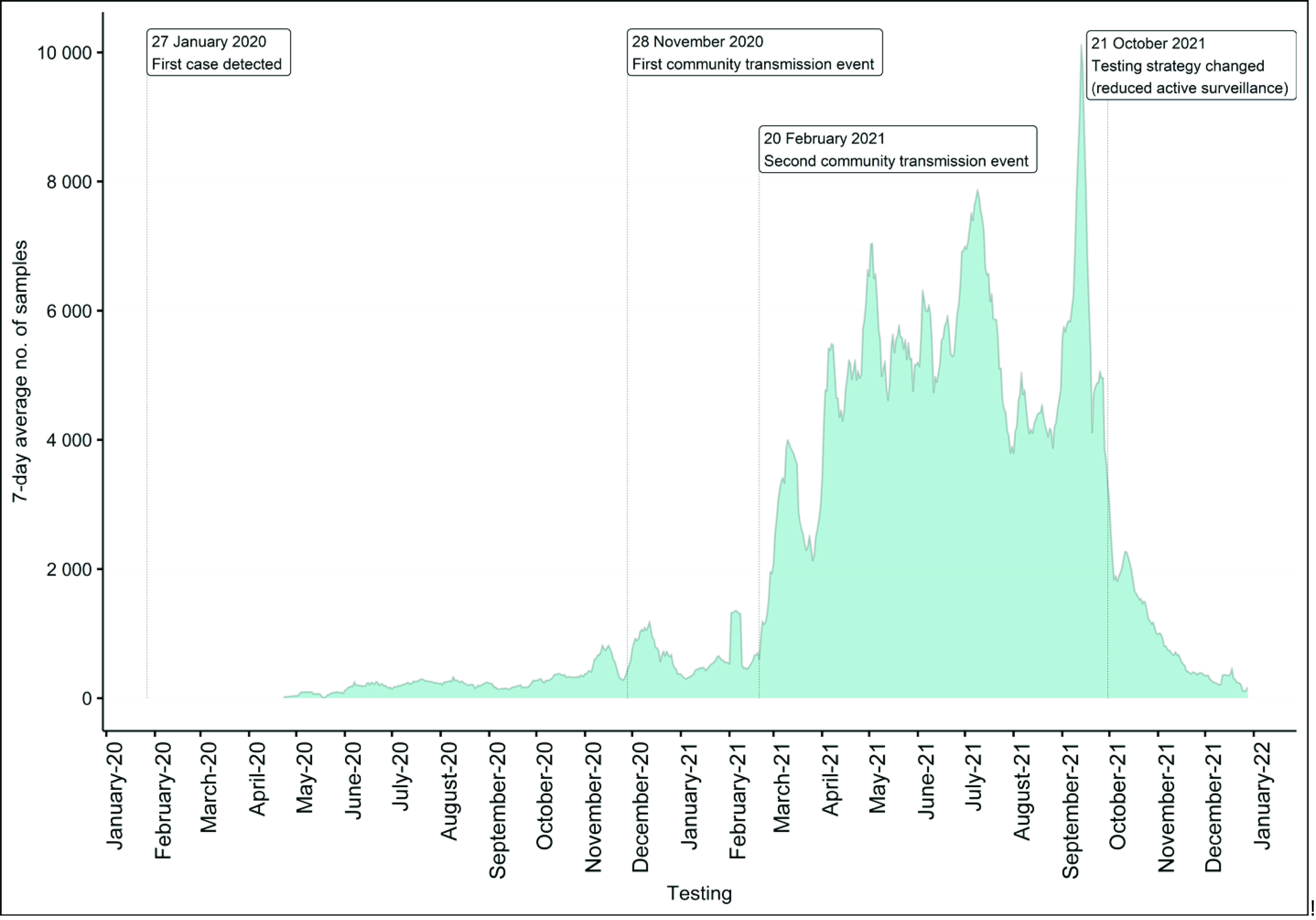
Number of samples tested daily for COVID-19 at the National Institute of Public Health, Cambodia, 2020–2021

At the beginning of the pandemic, Cambodia heavily relied on the nongovernmental testing capacity available at the Institut Pasteur du Cambodge. NIPH’s laboratory had to be set up and scaled up rapidly. Of the 2 575 391 COVID-19 tests conducted in Cambodia during 2020 and 2021, NIPH conducted 1 294 016 (50.2%; **Fig. 1**), while the Institut Pasteur conducted 827 613 (32.1%) and the other 18 laboratories conducted 453 762 (17.6%).

[**insert** Figure 1]

NIPH also provided technical advice during the establishment of 18 additional laboratories in  Phnom Penh and 11 other provinces about nucleic acid amplification tests (e.g. PCR, Cepheid’s GeneXpert platform and the Roche cobas test). (*5*) NIPH supported five essential elements under Pillar ii: assessing provincial laboratories’ quality management systems to ensure that all target laboratories could maintain good-quality testing services; providing training for provincial laboratory staff in PCR testing (i.e. training between 3 and 10 staff in PCR techniques and approximately 5 more staff to conduct related work, such as data entry and basic data management); providing guidance to provincial laboratory staff to ensure that appropriate infrastructure, equipment and supplies for performing PCR testing were available; establishing each provincial laboratory’s information management system to ensure that results could be appropriately recorded and reported to the Ministry of Health; and providing guidance to provincial laboratory staff to ensure that the PCR testing process was verified correctly.

### Pillar iii: surveillance

The CDCD managed the COVID-19 surveillance system with support from NIPH and other institutions. To support this pillar, NIPH undertook three activities.

First, NIPH tested the specimens collected through the national severe acute respiratory infection (SARI) and influenza-like illness (ILI) surveillance systems. (*6*) These specimens were tested for SARS-CoV-2 and influenza. Between 1 January 2020 and 31 December 2021, NIPH tested a total of 6706 of these specimens: 4636 (69.1%) were SARI and SARS-CoV-2 specimens and 2070 (30.9%) were ILI and SARS-CoV-2 specimens.

Second, NIPH collaborated with the Samdech Techo Voluntary Youth Doctor Association (TYDA) to establish COVID-19 testing sites throughout Phnom Penh. In 2020 and 2021, these sites collected 852 137 specimens, accounting for 33.1% of all specimens collected in the country.

Last, NIPH formed a COVID-19 data team that produced weekly reports for timely dissemination to several key stakeholders involved in the response. These reports included epidemiological data (i.e. the numbers of cases and deaths, specifying person, place and time); laboratory data (i.e. the number of samples tested and positivity rates, by district and commune); and other dynamic content (i.e. vaccine effectiveness rates, case forecasts, case reproduction numbers [the estimated number of secondary cases caused by a primary case] and proportions of different COVID-19 strains among positive samples). NIPH had produced 31 reports by  30 April 2022.

### Pillar iv: points of entry

Up until 31 December 2021, NIPH in collaboration with TYDA collected specimens from travellers at Phnom Penh International Airport and trained 238 trainers in border management for COVID-19. The training covered specimen collection, infection prevention, and fundamentals of isolation and quarantine.

### Pillar v: rapid response teams

By the time Cambodia detected its first COVID-19 case on 27 January 2020, the CDCD had enlisted more than 2000 rapid response team members. (*7*) Between April and July 2020, NIPH in collaboration with the CDCD trained 504 trainers in COVID-19 responses and contact tracing (additional staff were also trained, but NIPH was not involved).

### Pillar vi: infection prevention and control

NIPH experts were coauthors of two guidelines about infection prevention and control: the standard operating procedures for COVID-19 vaccination and the guidelines for prevention and control of COVID-19 transmission at health facilities and clinics.

### Pillar vii: case management and continuity of essential services

In 2020, NIPH in collaboration with other partners provided 26 trainings to 1497 trainers, both health centre staff and volunteers, about caring for and treating patients with COVID-19. Training partners included the Ministry of Health’s Department of Hospital Service, the CDCD, the University of Health Sciences, Calmette Hospital, Khmer–Soviet Friendship Hospital and the Cambodia–China Friendship Preah Kossamak Hospital.

### Pillar viii: logistics, procurement and supply management

In Cambodia, this pillar focused on (a) strengthening supply chains and the distribution of COVID-19 commodities, test kits and other material for testing; and (b) mobilizing resources from both domestic and development partners.

In 2020, NIPH estimated the need for materials and developed distribution strategies using a COVID-19 emergency loan and grants from the World Bank, the Global Fund and other agencies. In July 2020, NIPH successfully applied for a World Bank grant of more than US$ 1 million to purchase equipment and materials to set up the new laboratories for PCR testing in Phnom Penh and 11 provinces. After the laboratories were set up, NIPH received reports on the usage of the test kits and materials, as well as requests for additional kits and materials. For the Ministry of Health, the NIPH also estimated the need for test kits and materials among all public laboratories.

In 2021, NIPH also prepared testing packages for mobile testing teams that were used to collect 735 396 specimens in Phnom Penh.

### Pillar ix: risk communication and community engagement

NIPH did not contribute to any risk communication and community engagement activities. These were coordinated through the Subcommittee for Education, Training and Public Affairs, with strong organizational and financial support from partners. (*8*)

## Discussion

During the first 2 years of the COVID-19 response in Cambodia, NIPH contributed to eight of the nine pillars adapted from WHO’s 2020 strategic preparedness and response plan. NIPH’s main contributions were to support laboratory services and incident management and planning.

The NIPH laboratory tested half of the total 2 575 391 COVID-19 samples collected in Cambodia during 2020 and 2021 and provided technical advice for the establishment of 18 additional laboratories for SARS-CoV-2 testing across the country. NIPH’s laboratories did not expect to do so much of the testing. Before the pandemic, NIPH tested only a few hundred SARI and ILI specimens per week. By July 2021, NIPH had increased its capacity from 100 PCR tests per day in April 2020 to 6000 PCR tests per day. In September 2021, during a few extreme days, NIPH performed 10 000 PCR tests per day. Investing in a network of high-quality governmental laboratories was vital to address the current COVID-19 crisis, and these will be important assets for future emergency responses.

NIPH participated in policy development and provided advice by having representation on six national COVID-19 response subcommittees, as well as on the High-level Ministry of Health Task Force, the Technical Working Group and the Committee for Vaccination. NIPH’s Health Systems Research and Policy Support Center and the School of Public Health had increased the Institute’s capabilities in research and policy development before the pandemic, which enabled it to make contributions to the subcommittees. Investments in NIPH should be sustained to consolidate and further strengthen public health capacities and readiness in the country.

This is the only report that describes NIPH’s contribution to the COVID-19 response in Cambodia from the perspective of those from the Institute who implemented the response. However, the report has limitations. First, as most authors are NIPH staff, the report may be biased towards the Institute. Second, the contributions made by other institutions to the COVID-19 response, such as those of the Institut Pasteur du Cambodge and the CDCD, have not been included. Finally, the overall governance and collaboration of all relevant institutions during the COVID-19 response was also not assessed.

The role of NIPH was essential to the COVID-19 response in Cambodia, particularly in providing laboratory services and technical advice and in contributing to policy development through membership of its staff on national committees. Based on the contributions made by NIPH during the COVID-19 pandemic, continued investment in the Institute is critical to enable it to provide support during future health emergencies in Cambodia.
